# Is Mn(I) More Promising Than Fe(II)—A Comparison of Mn vs Fe Complexes for Olefin Metathesis

**DOI:** 10.1021/acs.organomet.3c00398

**Published:** 2024-02-05

**Authors:** Jan Pecak, Radu A. Talmazan, Dennis Svatunek, Karl Kirchner, Maren Podewitz

**Affiliations:** † Institute of Materials Chemistry, TU Wien, Getreidemarkt 9, Vienna 1060, Austria; ‡ Institute of Applied Synthetic Chemistry, TU Wien, Getreidemarkt 9, Vienna 1060, Austria

## Abstract

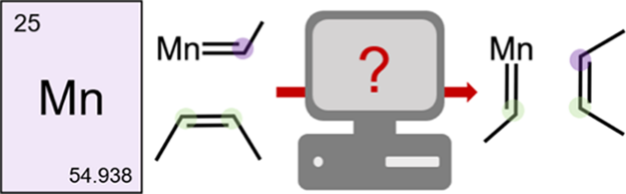

Olefin metathesis is one of the most significant transformations in organic chemistry and is an excellent example for efficient homogeneous catalysis. Although most currently used catalysts are primarily based on 4d and 5d metals, cycloaddition and cycloreversion reactions can also be attributed to first-row transition metals, such as Fe. Surprisingly, the potential of Mn(I)-based catalysts for olefin metathesis has been unexplored despite their prominence in homogeneous catalysis and their diagonal relationship to Ru(II). In the present study, we have investigated the prospective capabilities of Mn complexes for cycloaddition and reversion reactions using density functional theory. Therefore, we have initially compared the literature known iron model systems and their isoelectronic Mn counterparts regarding their reactivity and electronic structure. Next, we constructed potential Mn complexes derived from synthetically accessible species, including carbonyl ligands and obeying octahedral geometry. Based on thermodynamic parameters and the calculation of electronic descriptors, we were able to validate the isodiagonal relationship. Our study serves as guidance for the experimental chemist.

## 1 Introduction

**Figure 1:**
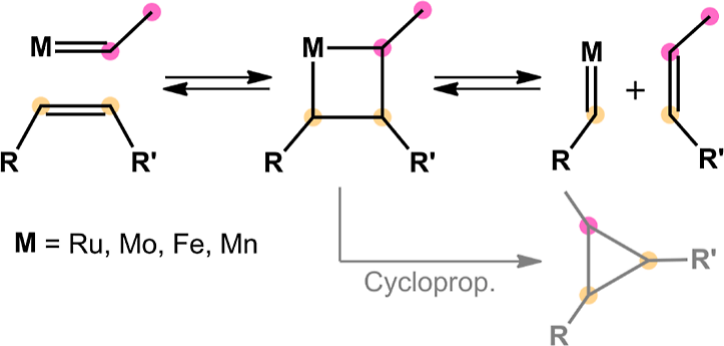
Olefin metathesis according to Chauvin’s mechanism including cyclopropanation, the undesired side reaction often encountered in first-row TM complexes.

Metathesis reactions are among the most important chemical processes in modern organic synthesis. They are used to efficiently break and rearrange carbon–carbon bonds obeying excellent atom economy, both in industrial-scale applications, as well as in chemical and pharmaceutical research.^1^ From a mechanistic perspective, olefin metathesis (OM) is based on a [2 + 2]-cycloaddition/-reversion cycle that is catalyzed by proficient and well-defined metal–alkylidene complexes involving a metallacycle intermediate. Cyclopropanation can be an undesired side reaction in this process (Figure 1). For their pioneering work in this very field, Chauvin, Schrock, and Grubbs were awarded the Nobel Prize in chemistry in 2005.^2−4^

Consequently, the most widely used types of catalysts for metathesis (of any type and flavor) are high oxidation state molybdenum complexes developed by Schrock and the ruthenium-based systems later developed by Grubbs. Important modifications to the existent systems and contributions to this field have been reported by Hoveyda^5^ and Copéret,^6,7^ as well as Buchmeiser,^8,9^ Blecher,^10^ and Nolan.^11,12^ Even though the current catalysts provide high yields under mild conditions, the development of sustainable, nontoxic, and cheap base-metal catalysts is highly desirable.^13^ One area of research that is currently of significant interest is the substitution of ruthenium with iron, the corresponding 3d transition metal of the first series of the periodic table. Numerous research groups have been working on the design of operational iron systems for OM, both experimentally and theoretically.^14−23^ Within this context, the research groups of Bukhryakov and Milstein have recently reported on new low-valent iron complexes to be active in ring-opening metathesis polymerization.^24,25^ Recent advances regarding the chemistry of iron carbene complexes and iron-based cycloaddition can further be attributed to Illuc^26^ and Meyer.^27^

While iron is well accepted as a surrogate for ruthenium in OM, it is somehow surprising not to find any investigations or at least considerations on the use of manganese for the same purpose. In recent years, several catalytic transformations involving Mn(I) complexes have been reported to exhibit similar structure, reactivity, and stability as compared to established Ru(II) and Fe(II) systems.^28−32^ Due to similar chemical properties between elements of the second and third period, Ru(II) and isoelectronic Mn(I) can be assumed to constitute a diagonal relationship in the periodic table.^33,34^ This relationship is strongly inspired by the successful application of vanadium alkylidenes in OM, mimicking the reactivity of related Mo complexes.^35,36^ Unfortunately, the total number of currently known and stable manganese carbene complexes is small and mostly composed of Fischer-type complexes. In contrast to Mo and Ru-based catalysts, these compounds readily undergo cyclopropanation reactions.^37,38^ Fischer-type carbenes are defined as M=CRX with X being a heteroatom and are thus electrophilic by nature, in contrast to Schrock-type carbenes (M=CR_2_). The only Mn system that resembles elements of OM was reported by Braunschweig and co-workers in 2013 and involves the formation of [MnCp(CO)_2_(=CPh_2_)] via cycloreversion of a Mn borylene metallacycle.^39^ Consequently, manganese-catalyzed olefin metathesis has (in contrast to iron) been neither theoretically studied nor experimentally attempted before.

All approaches to use elements of the first transition metal series for olefin metathesis face certain intrinsic challenges that need to be addressed in order to devise an active catalyst: (i) the metal–carbene (M=CR_2_) bond tends to be weaker in 3d metal systems than in 4d/5d transition metal systems.^18,40^ This fact leads to cyclopropanation being the preferred reaction in first row transition metal (carbene) complexes.^41,42^ (ii) While heavier metals such as Ru and Mo favor low-spin closed shell configurations, first row metals usually exhibit a wide range of possible spin states, which are close in energy. (iii) Surface crossings between singlet and triplet states are likely to take place.^43^ (iv) Open shell systems hamper the coordination of an olefin and could very likely also undergo other radical reactions. However, in the absence of experimental data, theoretical studies can aid in guiding experimental synthesis, predicting trends and patterns, and excluding unsuitable systems. Due to the complex electronic structure and the multitude of accessible spin states in Mn (and Fe) systems, the computational chemist faces the problem of accurately describing these states and their relative energies.^44^ For these reasons, it is not surprising that the first Fe systems to show activity in OM were discovered years after their computational exploration. In this contribution, we explore the feasibility of low-valent manganese alkylidene species complexated by mono-, bi-, and tridentate ligand systems for potential use in OM by means of density functional theory. The focus is on the comparison of isostructural Fe/Mn systems as well as on the conception of systems that are building upon already synthetically viable precursors.

## 2 Results and Discussion

### 2.1 Comparison of Isoelectronic Fe and Mn Systems

**Figure 2:**
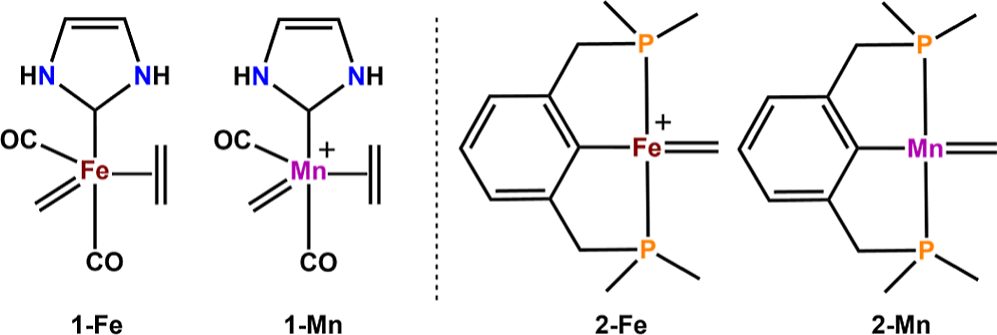
Literature-derived isoelectronic model catalysts for olefin metathesis.

In pursuit of an in silico operational manganese system for olefin metathesis, we developed a series of suitable model complexes that allow for a comparison with already known and investigated systems regarding spin state splittings, electronic structure, and thermodynamic parameters. Figure 2 displays two isostructural and isoelectronic model systems (**1-Fe**/**1-Mn** and **2-Fe**/**2-Mn**) derived from the existing literature.

For a direct comparison with a literature known Fe system, we first adopted the simple model catalyst **1-Fe** reported by Mauksch and Tsogoeva^22^ and substituted the putative Fe(II) center with formally isoelectronic Mn(I). The main focus of this 2017 publication was to demonstrate the possibility of low-valent iron for OM based on orbital symmetry and aromaticity arguments. For simplicity reasons, only one topological isomer of this system was considered, bearing the coordinated olefin, a CO ligand, and the carbene fragment in the equatorial plane. Second, we adopted a tridentate pincer-type carbene system with a benzene backbone (**2-Mn**) as devised by Solans-Monfort and co-workers.^16^ The latter work concluded that Fe=CH_2_ fragments and metallacyclobutanes are ideally stabilized in the singlet state utilizing ECE pincer ligands (E = NHC, PR_2_) with a strong σ-donor present in the central position.

For these model systems, relevant stationary points along the OM reaction path were calculated. This includes the initial olefin adduct, the corresponding metallacyclobutane structure, as well as the transition states (TS) for [2 + 2] cycloaddition, cycloreversion, and cyclopropanation for all relevant spin states. Since all model complexes exhibit a d^6^ electron configuration, a low-spin (S = 0) singlet state, a triplet state (S = 1), and a quintet high-spin (S = 2) state can be relevant. For calculations of the electronic structure and thermodynamic parameters, we adopted the meta-hybrid exchange–correlation functional TPSSh (with 10% HF exchange), as it was found to accurately predict spin state splittings of manganese and iron-based spin crossover compounds.^45,46^ Nevertheless, other density functionals were also used, and DLPNO-CCSD(T) calculations were performed on selected structures to gauge the accuracy of the methodology.

In order to gain a better understanding of the M=CH_2_ (M = Fe, Mn) bonds in our singlet model systems, we first carried out energy decomposition analysis (EDA-NOCV) as applied and advocated by Frenking and co-workers.^47,48^ The EDA decomposes the interaction energy Δ*E*_int_ between two fragments within a molecule into three different components, namely, Δ*E*_elstat_, Δ*E*_Pauli_, and Δ*E*_orb_, in order to obtain a chemically meaningful bonding picture. Thereby, either two closed-shell fragments that interact or two triplet fragments that interact in an antiparallel fashion can be generated in the EDA. Whichever fragmentation yields values closer to zero for the orbital mixing term Δ*E*_orb_ determines the chemical interpretation of the bonding. If the two singlet fragments yield a lower value, the bond is termed dative, whereas if it is the two antiparallel triplet fragments, the bonding is referred to as electron sharing. Metal carbene complexes with electron-sharing bonding are traditionally classified as Schrock-type (alkylidene) while those with a dative bonding as Fischer-type. Table 1 shows a compilation of the energy values obtained by a decomposition analysis with TPSSh/TZ2P/D3. Complex **1-Fe** can be described as electron-sharing bearing two interacting triplet fragments, while the carbene bond in **1-Mn** can rather be described with a dative interaction. However, the differences for the decisive Δ*E*_orb_ values in the manganese and iron systems are small. To further investigate the nature of the M=CH_2_ bond, Wiberg bond orders were calculated to be 1.70 in the Fe complex and 1.91 in the Mn complex, respectively. This analysis points to a more pronounced double bond character in the Mn system. According to EDA, the model catalysts **2-Fe** and **2-Mn** can both be described as having two interacting triplet fragments and can therefore be classified as Schrock-type carbenes. The associated bond order is 2.11 for the Fe system and 2.19 for the Mn system, respectively. Consequently, the double bond character in these complexes is more pronounced than those in **1-Fe** and **1-Mn**. In all cases investigated, the total interaction energy Δ*E*_int_ of the closed-shell fragments was much lower (negative) than that of the triplet fragments. Based on this limited data set, no clear differentiation of Fe vs Mn carbene complexes can be made. Within one pair of complexes Fe and Mn behave rather similar, while the differences attributed to the different ligand spheres are more pronounced between the two pairs, **1-Fe**/**1-Mn** vs **2-Fe**/**2Mn**. A comparison with related Ru-based carbene species, namely, **2-Ru**, the analogous structure to **2-Mn** and a second-generation Grubbs catalyst, revealed that these also favor an electron-sharing bonding mode. At least within the EDA, the carbenes can be considered as similar—highlighting once more the diagonal relationship of Ru and Mn. Details about these calculations can be found in the Supporting Information (Table S1).

**Table 1:** Results of the EDA of the Model Compounds **1-Fe**, **1-Mn**, **2-Fe**, and **2-Mn**^a^

	bonding mode	Δ*E*_int_	Δ*E*_elstat_	Δ*E*_orb_	Δ*E*_Pauli_
**1-Fe**	dative	–114.9	–176.8	–141.2	205.5
**1-Mn**	dative	–110.7	–198.5	–146.7	238.4
**2-Fe**	dative	–126.1	–242.2	–171.4	292.9
**2-Mn**	dative	–127.6	–220.7	–173.3	271.5
**1-Fe**	electron-sharing	–92.9	–124.0	–139.4	172.9
**1-Mn**	electron-sharing	–95.8	–113.7	–149.4	171.2
**2-Fe**	electron-sharing	–78.4	–119.1	–155.1	201.2
**2-Mn**	electron-sharing	–86.4	–122.7	–157.9	199.2

a Dative bonding results from the interaction of two singlet fragments, whereas electron-sharing bonding results from triplet–triplet interactions. All energies are given in kcal/mol obtained using TPSSh/TZ2P/D3.

**Figure 3:**
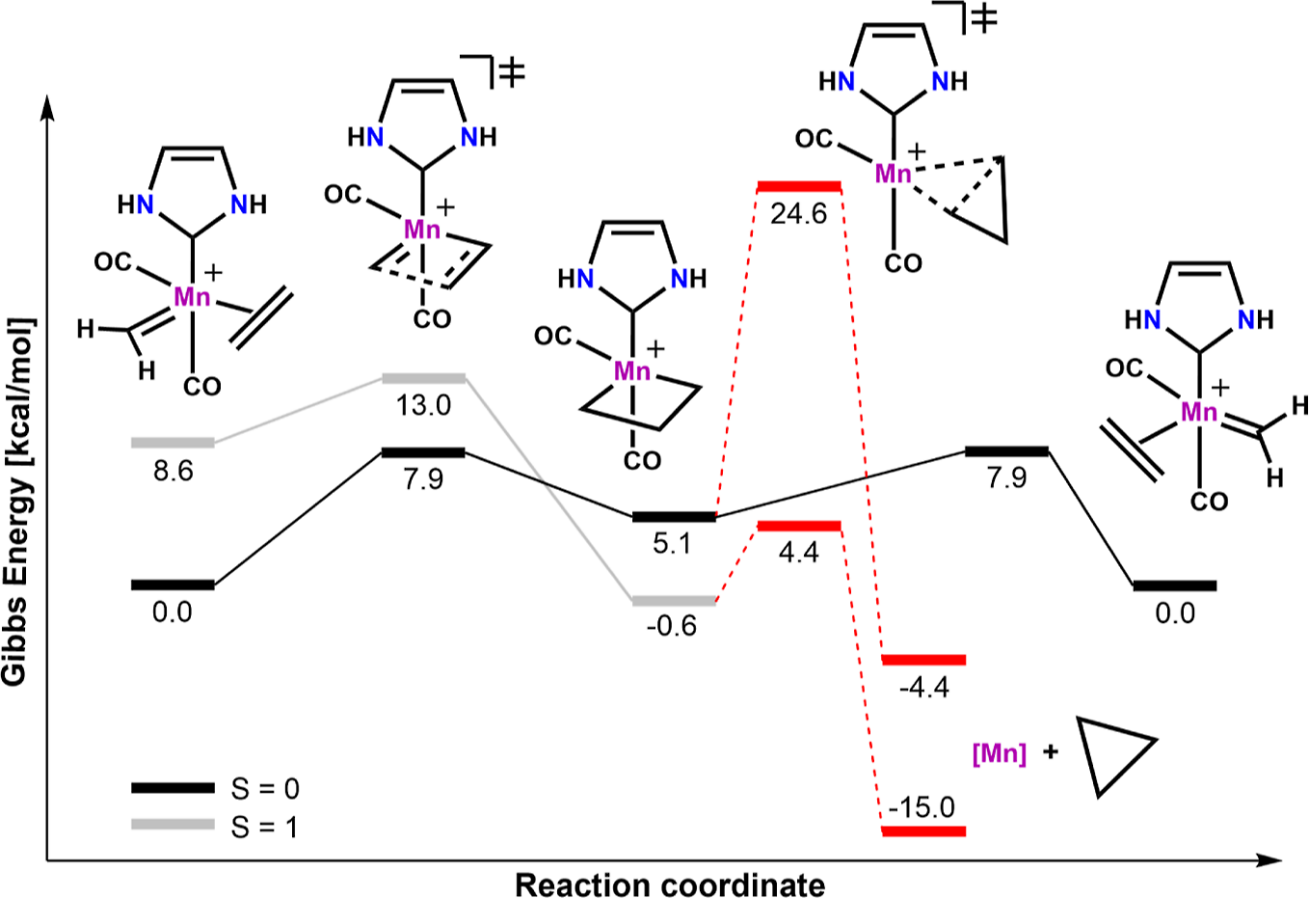
Reaction energy profile for OM with model catalyst **1-Mn**. The reaction paths describing cyclopropanation are depicted with dotted lines and a red color. For the sake of simplicity, the cycloreversion step in the triplet state is omitted as it is symmetric to the cycloaddition. All energies are given in kilocalories per mole obtained using TPSSh/def2-TZVP/D3//TPSSh/def2-SVP/D3 in dichloromethane.

The reaction energy profile for the simple model catalyst **1-Mn** with a Grubbs-type N-heterocyclic carbene (NHC) moiety is depicted in Figure 3. The starting point in this olefin metathesis cycle is the stable olefin adduct (technically the first intermediate) that results from the coordination of the olefin to **1-Mn**. As opposed to the reported Fe system, it can be observed that the Mn=CH_2_ unit in the optimized closed-shell olefin adduct is rotated by almost 90°, which results in a noncollinear arrangement with the coordinated olefin (see Supporting Information, Figure S1). Moreover, there is a substantial deviation from the ideal trigonal bipyramidal arrangement of the ligands. These changes in geometry resemble the reported Fe triplet geometry instead of the anticipated singlet one. Additionally, it was found that the calculated M-C_α_ bond lengths in the optimized singlet Mn metallacycle are 0.10 Å shorter than for the Fe case. The singlet metallacycle displays a trigonal bipyramidal geometry, while the triplet geometry resembles a distorted square pyramid. The metallacyclobutane formation is also slightly endergonic with 5.1 kcal/mol, but the metathesis process is feasible and clearly preferred over cyclopropanation, as indicated by a difference of Δ*G*(TS_cycloprop_) (24.6 kcal/mol) and Δ*G*(TS_cyclorevers_) (7.9 kcal/mol) of 16.7 kcal/mol. Furthermore, the initially mentioned rotation of the Mn=CH_2_ unit is likely responsible for the slight increase in the energy of the consecutive TS for [2 + 2]-cycloaddition as compared to the iron congener. For comparison with literature (DFT/OPBE), OM with the active Grubbs catalyst [Ru(IMesH_2_)(Cl_2_)(=CH_2_)] (IMesH_2_ = 1,3-dimesityl-imidazole-4,5-dihydro-2-ylidene) displays a barrier of 12.7 kcal/mol for cycloaddition of ethylene and a barrier of 37.7 kcal/mol for decomposition via cyclopropanation, hence a larger separation of the two pathways.^16^

Unlike, Ru complexes, the singlet–triplet energy splitting for the metallacyclobutane intermediate is one of the most crucial parameters in first-row TM metathesis and for **1-Mn** was calculated to be Δ*G*_ST_ = −5.7 kcal/mol with TPSSh and Δ*G*_ST_ = −5.4 kcal/mol employing DLPNO-CCSD(T) in favor of the triplet state. A survey on the performance of various exchange–correlation functionals on the singlet–triplet splitting for metallacycle **1-Mn** can be found in the electronic Supporting Information (see Table S2). Essentially all of the tested functionals yielded similar trends. Coming back to **1-Mn**, the quintet state of the metallacyclobutane is located just 0.4 kcal/mol above the triplet state and could also play a role in surface crossing events and deactivation processes. Similar to the reported iron system (**1-Fe**), the TS for the [2 + 2]-cycloaddition in the singlet state also exhibits aromatic character with a NICS(1) value (NICS = nucleus independent chemical shift) of −18.7 ppm (cf. −19.4 ppm) which further supports our initial assumption on the isodiagonal relationship between Ru(II)/Fe(II) and Mn(I) (vide supra).^49^

Next, we investigated the reactivity and reaction energetics of **2-Mn**, where Mn is coordinated by an anionic PCP^50,51^ pincer-type ligand. Similar to the iron CCC, PCN, and CCN pincer complexes investigated by Solans-Monfort, the M=CH_2_ unit in the initial carbene complex **2-Mn** (as depicted in Figure 2) is directed away from the PCP plane at an obtuse angle. In the triplet state, **2-Fe** and **2-Mn** both have almost ideal square planar geometry. In sharp contrast to the reported Fe systems, (in the absence of a coordinated olefin) already the initial carbene complex **2-Mn** in the triplet state is 20.5 kcal/mol more stable than that in the envisioned singlet state. This finding is also supported by OPBE calculations with an Δ*G*_ST_ value of −10.3 kcal/mol.

**Figure 4:**
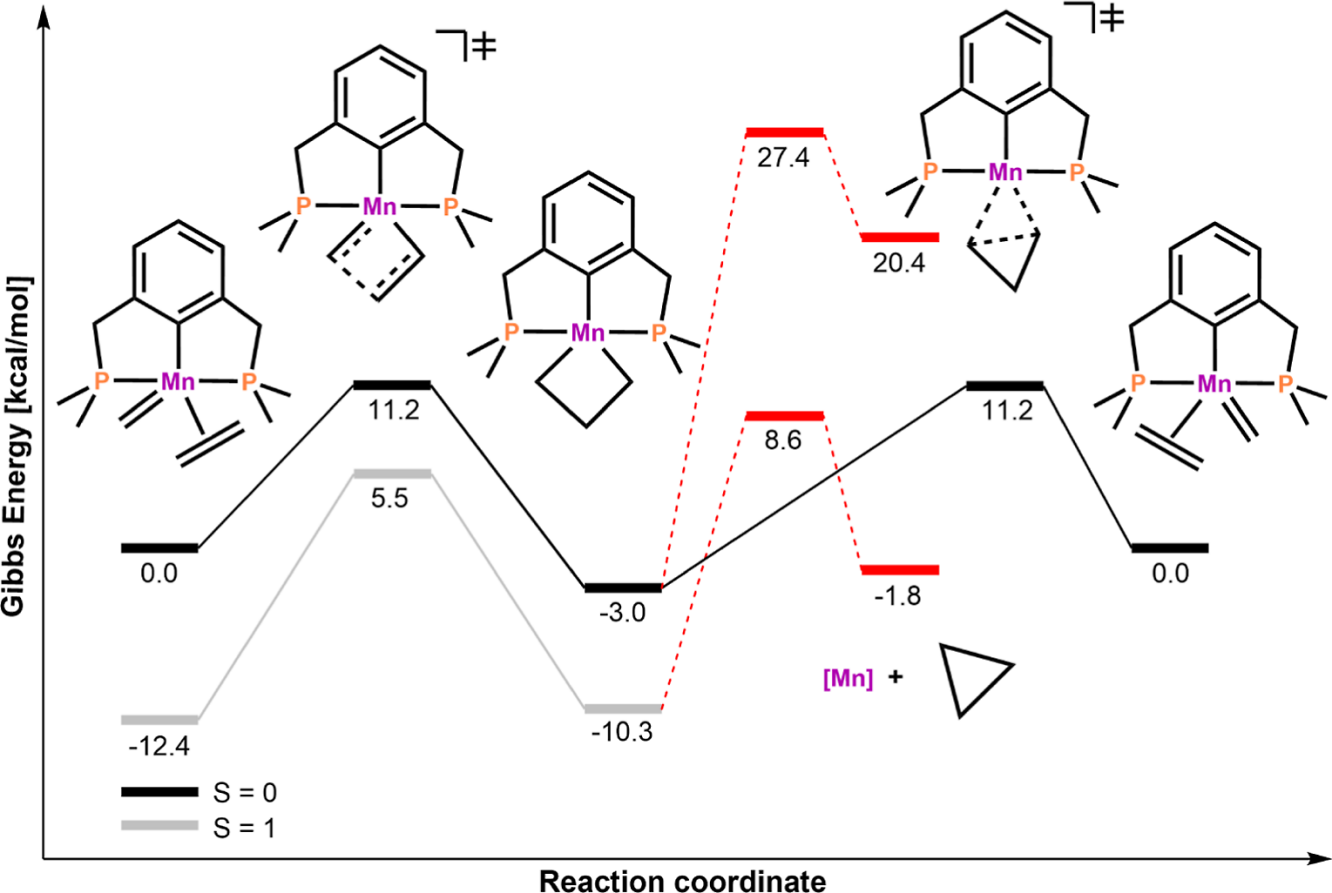
Reaction energy profile for OM with the model catalyst **2-Mn**. The reaction paths describing cyclopropanation are depicted with dotted lines and red color. Note that the starting point in this figure is already the coordinated olefin adduct. For the sake of simplicity, the cycloreversion step in the triplet state is omitted as it is symmetric to the cycloaddition with a TS energy of 5.5 kcal/mol. All energies are given in kcal/mol obtained using TPSSh/def2-TZVP/D3//TPSSh/def2-SVP/D3 in dichloromethane.

The reaction energy profile for olefin metathesis with **2-Mn** is shown in Figure 4. Again, the depicted reaction profile starts with the olefin adduct that can be generated by coordinating an olefin to **2-Mn**. The formation of the olefin adduct from the separated singlet reactants is favored by Δ*G* = −20.4 kcal/mol (not displayed), while the olefin adduct is more stable in the triplet state than in the singlet state. The energetic barrier for the initial [2 + 2]-cycloaddition on the singlet surface is 11.2 kcal/mol and leads to the formation of a singlet metallacycle with a trigonal bipyramidal geometry. Similar to the situation of **1-Mn**, the metallacyclobutane intermediate is more stable in the triplet state than in the singlet state with Δ*G*_ST_ = −7.3 kcal/mol. This trend is again supported by OPBE calculations, yielding a Δ*G*_ST_ value of −12.9 kcal/mol. The barriers for cyclopropanation were calculated to be 30.4 kcal/mol in the singlet state and 18.9 kcal/mol in the triplet state, respectively, and are higher in energy than productive OM. In addition, the total cyclopropanation reaction is an endergonic process for both spin states. Note that cycloaddition and cycloreversion are symmetry-related processes in this specific example, and therefore, starting points and end points, as well as corresponding TSs, have the same energy (Figure 4: cycloreversion for the S = 1 state is omitted as it is also 5.5 kcal/mol). We point out that, despite the shortcomings of the system, the metathesis process is preferred over cyclopropanation in both the singlet and triplet states, which is an important necessity for OM. This also constitutes a major difference to iron-based systems such as **2-Fe**, where the triplet state inevitably leads to decomposition. As with **1-Mn**/**1-Fe**, also in the case of **2-Mn**/**2-Fe**, the spin-state ordering in the Mn variant is less sensitive to the chosen density functional than that in the Fe counterpart.

**Figure 5:**
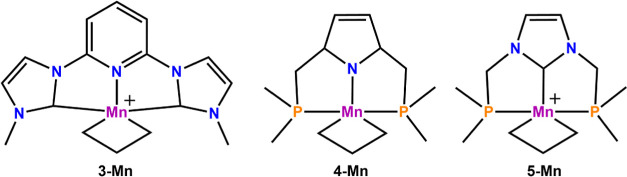
Extended test set of Mn metallacyclobutane complexes with pincer-type ligands.

To further investigate the influence of the donor strength and design of the supporting pincer ligands, we considered three additional frameworks. As for the OM, the metallacyclobutane structure is the key intermediate and should ideally be in the singlet state. Hence, we investigated the impact of these ligands on Δ*G*_ST_ in the corresponding metallacyclobutane complexes (see Figure 5). For **3-Mn** a singlet–triplet splitting of −18.3 kcal/mol was found, for **4-Mn** a splitting of −7.2 kcal/mol and for **5-Mn** a value of −8.6 kcal/mol, respectively. In all of these cases (including **2-Mn**, vide supra), the triplet state remains the more favorable one, but a strong influence of the ligands can be observed. Similar to the computational studies of iron complexes, the effect of the central donor is pivotal, but the stabilization of the singlet state in Mn systems is much more difficult to achieve than in related Fe systems at least based on the limited existing data. It is also particularly noteworthy at this point that the deviations from ideal ⟨S^2^⟩ in the calculated triplet states are significantly higher for the calculated Mn pincer species than for the corresponding Fe pincer species, indicating a more complex electronic structure of the Mn systems. Earlier investigations by Chirik and co-workers already revealed the complexity of the electronic structure of pincer-coordinated Fe carbene complexes.^52^ It would certainly be of interest to examine the influence of various pincer ligands and substituent effects^53^ in more detail, but this is beyond the scope of this current article.

In addition to the consideration of purely thermodynamic and kinetic parameters, electronic descriptors such as the NMR chemical shift (tensor) can also be used to describe activity in olefin metathesis. Gordon et al. derived from solid-state ^13^C NMR experiments and DFT calculations that active metallacyclobutanes usually have isotropic shifts for C_α_ and C_β_ of 80–100 ppm and <10 ppm, respectively.^54^ Applying this concept to the metallacyclobutane structure derived from **1-Mn**, we computed δ(α) = 119 ppm and δ(β) = −11 ppm. A similar and even better result is obtained for **2-Mn** with δ(α) = 115 and δ(β) = 4 ppm. Even though these rules were primarily derived from Ru, Os, Mo, W, and Ir complexes, the values obtained for both model complexes were found to match well and are in line with our assumption of an isodiagonal relationship.

Lastly, surface crossings between singlet and triplet surfaces must not be neglected for the investigated complexes and require closer inspection. A well-known concept in this regard is the use of minimum energy crossing points (MECP) to approximate the adiabatic transition between two spin states. Such processes are formally spin-forbidden in the absence of spin–orbit coupling. As suggested by Harvey, these points can be calculated using an optimization algorithm utilizing combined gradients for the singlet and triplet PES to eventually yield a geometry where Δ*E*_ST_ = 0.^55^ As mentioned before, our simple test system **1-Mn** (see Figure 3) displays a metallacyclobutane intermediate, with the triplet state being 5.7 kcal/mol more stable than the singlet state. We could localize a MECP at Δ*G*_MECP_ ≈ 2.3 kcal/mol above the singlet state, low enough to allow thermal deactivation of the catalyst by surface crossing. Similarly, for the PCP complex **2-Mn** (see metallacycle in Figure 4) a MECP could be localized at Δ*G*_MECP_ ≈ 2.8 kcal/mol above the singlet state. These surface crossing points are easily accessible at reaction conditions and can compete with all other reaction steps.

### 2.2 Synthetically More Feasible Model Systems

**Figure 6:**
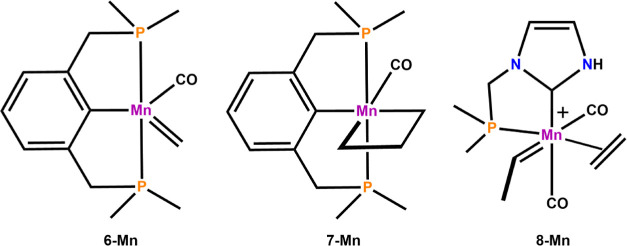
Modifications of the initial model catalysts by addition of CO and by inclusion of octahedral geometry.

While it is well-known that present OM catalysts in their active form are usually 4-fold coordinate species with a low d-electron count,^6^ even simple base-metal model systems such as **1-Mn** or **2-Mn** are very challenging to realize experimentally. The preparation of low-spin Mn(I) complexes is commonly based on the reaction of a suitable carbonyl precursor, such as Mn(CO)_5_Br or Mn_2_(CO)_10_ with bidentate or tridentate ligands and subsequent chemical modifications. Moreover, chemical experience dictates that the presence of carbon monoxide is an integral component of most low-spin manganese complexes and that a coordination number of five or six is usually encountered in these molecules.^56−58^ Even though CO is usually not found as a coligand in active OM catalysts, it is worth exploring these possibilities computationally since CO ligands can be helpful in maintaining a singlet ground state throughout a catalytic cycle. Figure 6 depicts a conceptual extension of the presented complexes **1-Mn** and **2-Mn**.

A possible starting point for a synthetically feasible compound such as **6-Mn** could be a Mn(I) dicarbonyl complex [Mn(PCP)(CO)_2_], similar to that described by Tonzetich and co-workers.^59^ Depending on the stability of the depicted system under experimental conditions, either a direct reaction and formation of an olefin adduct or metallacycle (**7-Mn**) or alternatively loss of remaining CO to eventually form a 4-fold coordinate complex such as **2-Mn** (vide supra) seems possible. The electronic effect of including a CO ligand in carbene complex **6-Mn** is clearly expressed in the stabilization of the singlet state and a Δ*G*_ST_ value of 8.1 kcal/mol. Note that the corresponding complex without CO is favored in the triplet state. However, an adverse effect is observed for the singlet–triplet splitting in the metallacycle **7-Mn**: a 6-fold octahedral coordination imposes strong geometrical constraints on the metallacycle and forces the metallacycle to adopt a distorted square pyramidal geometry rather than a trigonal bipyramidal coordination as observed naturally for **1-Mn** and **2-Mn** in the singlet state. As a consequence, the singlet state is destabilized, in comparison to the triplet state. Accordingly, a singlet–triplet splitting of −6.3 kcal/mol was obtained in favor of the triplet state for the metallacyclobutane complex **7-Mn**. As already outlined in the previous section, a singlet ground state for this important intermediate is a necessary prerequisite for OM.

In order to circumvent this problem and at the same time maintain the 6-fold coordination, bidentate ligands can be used. In this respect, **8-Mn** represents an octahedral modification and extension of **1-Mn**. In contrast to tridentate ligands, bidentate ligands can offer more structural flexibility and are less rigid. Experimentally, a compound such as **8-Mn** could be derived from a carbonyl precursor [Mn(NHC-P)(CO)_3_R] (R = Alkyl, Aryl, OAc) or a putative dinuclear precatalyst.^32,60^ To further increase the donor strength of the ligand, a bis-NHC system could be employed, which is also well-established in literature.^61^

**Figure 7:**
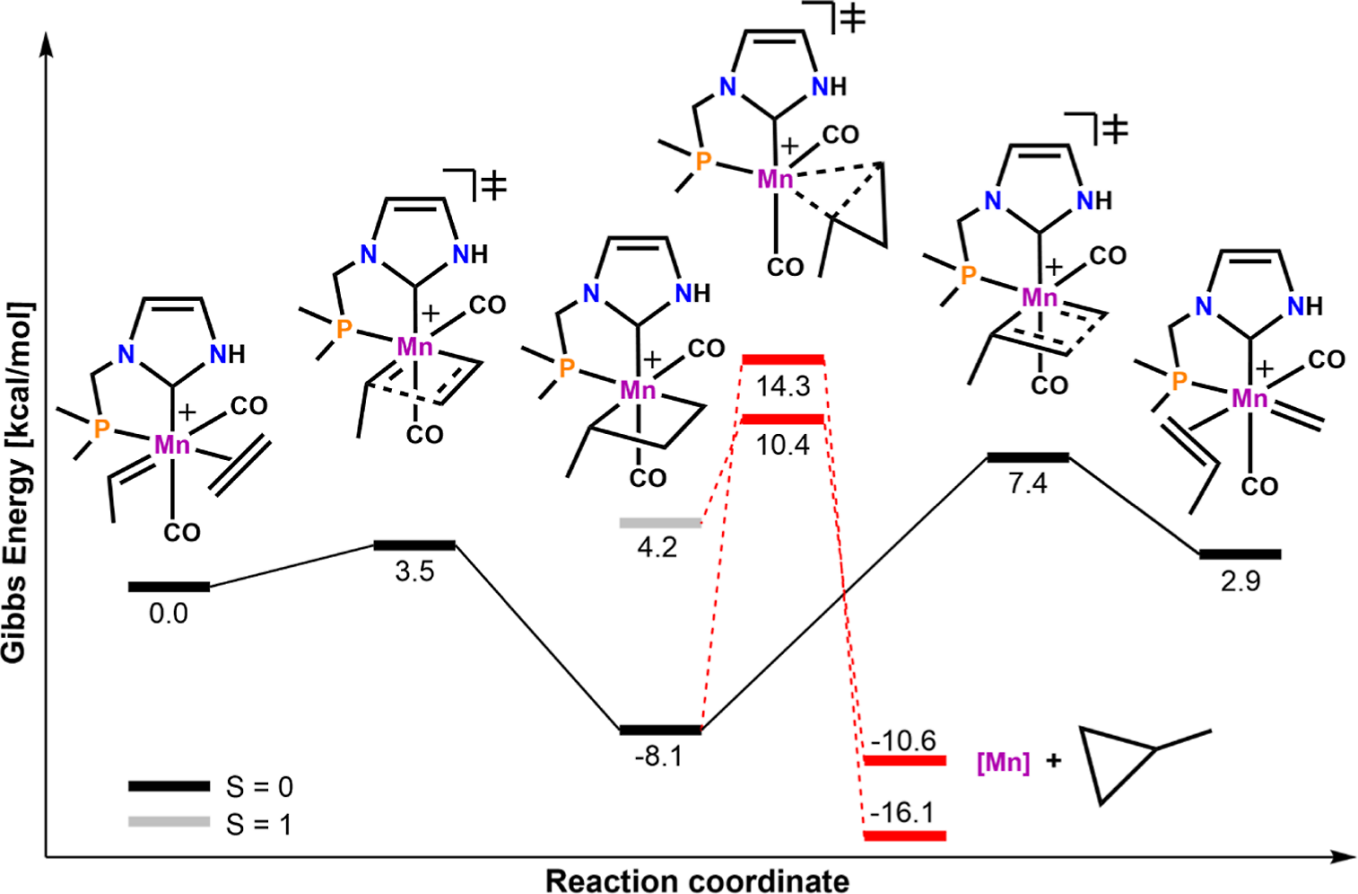
Reaction energy profile for OM with **8-Mn**. The reaction paths describing cyclopropanation are depicted with dotted lines and red color. All energies are given in kcal/mol obtained using TPSSh/def2-TZVP/D3//TPSSh/def2-SVP/D3 in dichloromethane.

The reaction energy profile for an olefin metathesis cycle with octahedral species **8-Mn** is shown in Figure 7. For simplicity reasons, again only one isomer was considered in the following and the starting point is the coordinated olefin adduct. The energetic barrier for the initial [2 + 2]-cycloaddition of the simple test substrate ethylene is low (Δ*G*^‡^ = 3.5 kcal/mol) and quickly leads to the formation of a stable singlet metallacycle. In the absence of surface crossings and isomerization reactions, the most important side reaction is cyclopropanation and the formation of methylcyclopropane. The barrier for this process was calculated to be 22.4 kcal/mol in the singlet state. The cycloreversion process forming the propene adduct at 2.9 kcal/mol displays a barrier of Δ*G*^‡^ = 15.5 kcal/mol. Note that educts and products are not identical and no symmetry relations are present anymore. The most important observation is the inversion of the spin state ordering (Δ*G*_ST_ > 0) and the preference for the singlet state of the metallacycle compared to **7-Mn**. This can be primarily attributed to the presence of the strong π-acceptor ligand CO as an additional ligand but also to the changes in electronic structure induced by altering the ligand sphere. Consequently, the effect of the CO ligands is 2-fold: it stabilizes the system in the singlet state, and the octahedral coordination prevents structural relaxation that is observed as a deactivation channel in 5-fold coordinated species such as **1-Mn** and **2-Mn**. To achieve the desired stabilization of the singlet state, an intricate balance of ligands is required.

**Figure 8:**
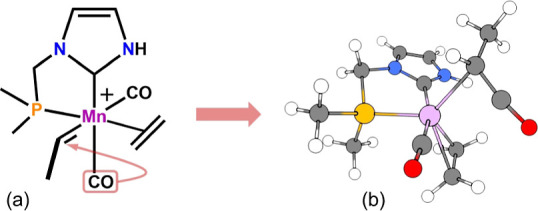
Migratory insertion like rearrangement reaction of carbonyl complex **8-Mn** (a) in the triplet state to a ketene-like system (b).

The incorporation of carbon monoxide into the chemical structure has fundamental advantages but also potential downsides. Regarding the geometry of olefin adduct **8-Mn** in the triplet state, we observed that the obtained minimum is 22.1 kcal/mol higher in energy. However, small displacements led to significant structural rearrangements and CO migration to the carbene (Figure 8). A similar optimization behavior was also found using other exchange–correlation functionals. Hence, it is not an artifact of the approximated DFT functional. This very stable species exhibits a Mn–C(H)(CH_3_)(C=O) motif and is located 2.0 kcal/mol above the singlet olefin adduct. It should not go unmentioned that migratory insertion of highly nucleophilic ligands such as alkyl groups is also a well-known concept in manganese carbonyl chemistry in the ground state.^60^ The quintet states for the adduct and the metallacycle showed dissociative character with respect to ethylene or CO, and thus could not be fully converged. We assume that both observed behaviors, the migratory insertion-like reactivity of the nucleophilic alkylidene in the triplet state and the dissociation of CO or olefin in higher spin states, could lead to early deactivation of the catalyst or precatalyst. Another potential side-reaction could be insertion of CO into the metallacycle, forming a metallacyclopentanone species. However, we have not observed such a behavior in the singlet or triplet states of our investigated Mn complexes.

## 3 Conclusions

The present work investigated the potential of olefin metathesis with a series of Mn-based complexes by exploring thermodynamics and energetics of homo metathesis with DFT. In the course of our investigation, we first compared isoelectronic iron and manganese complexes and later extended our test set to potentially more synthetically accessible carbonyl complexes. Our study highlights challenges that have to be addressed in the design of functional catalysts. Based on our findings, we conclude the following: (i) the overall thermodynamics and free energy profiles for the metathesis cycle with Mn-based systems are feasible and comparable to literature known Fe systems. (ii) Cyclopropanation is a viable side reaction in iron and manganese-based systems with the associated barriers being lower in the triplet state than in the singlet state. (iii) Similar to iron, control of spin states plays a crucial role in the conception of manganese catalyst for metathesis. (iv) Surface crossings are likely to take place if the triplet state is lower in energy. Together, this supports our assumptions on the isodiagonal relationship and the suitability of low-valent manganese systems, as they are comparable to iron. Our investigated complexes **1-Mn** and **2-Mn** do fulfill several requirements for OM such as low barriers for cycloaddition and -reversion, as well as feature electronic descriptors that are indicative for functioning OM—given a singlet spin state.

One aim of our investigation was to design systems that could be prepared in a laboratory and are feasible from a synthesis point of view. Mn pincer-type complexes or bidentate systems are well-known to the literature and known to stabilize Mn(I). They offer great variability in terms of their electronic properties and steric demand. Therefore, a very careful choice of pincer ligand could be sufficient to maintain a singlet ground state. However, based on our findings bidentate systems with additional CO (such as **8-Mn**) seem to be better suited to stabilize the singlet ground state.

Despite these yet to be tackled issues and the lack of experimental benchmark data, the high abundance of first-row transition metals provides an opportunity to develop inexpensive and nontoxic catalysts. Our research provides insights into the reactivity of Mn complexes and functions as a reference for the experimental chemist to pave the way for the design of manganese-based catalysts for olefin metathesis.^79^

## 4 Computational Methods

Computations in the present study were performed with the ORCA 5.0^62^ and ADF^63−65^ 2023.103 program package utilizing the Vienna Scientific Cluster (VSC 4) in part. Electronic ground state calculations, including geometry optimizations, frequencies, and transition-state searches, were carried out with density functional theory (DFT) utilizing ORCA and the TPSSh meta-hybrid functional^66^ together with Grimme’s D3 dispersion correction^67^ and Ahlrichs’ def2-SVP basis set.^68^ The resolution of identity approximation was used, along with the corresponding auxiliary basis sets, to accelerate the calculations. Gibbs free energies at 298 K were calculated using the rigid-rotor harmonic oscillator approximation as implemented in ORCA and real frequencies below 100 cm^–1^ were raised to 100 cm^–1^ to improve accuracy.^69^ Final single-point energies were calculated with TPSSh/def2-TZVP/D3 or OPBE/def2-TZVP/D3^70,71^ on already obtained geometries, while Gibbs energies were obtained by adding zero-point energies, thermal and entropic corrections at 298 K. For geometry optimizations and single-point calculations, implicit solvation was included via the CPCM model and dichloromethane as solvent.^72^ TSs were localized with the help of the nudged elastic band method as implemented in the ORCA and confirmed to be first-order saddle points by analysis of the Hessian. Intrinsic reaction coordinate calculations have been employed to verify the correct assignment of TSs by following the eigenvector of the corresponding imaginary frequency, starting from the TS structures and connecting to reactants and products. MECP were calculated using the algorithm implemented in ORCA and the SurfCrossOpt keyword.^73^ DLPNO-CCSD(T) calculations were performed on previously obtained TPSSh-geometries using the def2-TZVP basis set and a converged BP86^74^ UKS reference wave function.^75^ For the sake of completeness, it should also be mentioned that the wave functions of all Mn complexes were checked for internal stability: This revealed that the S = 0 species derived from **2-Mn** exhibit a slight instability toward an unrestricted Kohn–Sham (UKS) wave function. However, the electronic energy differences are negligible (<1 kcal/mol). EDA was specifically performed in ADF using the TPSSh functional together with the TZ2P basis set and no frozen core orbitals.^76^ The numerical quality was set to “very good”, and the scalar relativistic effects were accounted for using the zeroth-order regular approximation.^77^ For the interaction between triplet fragments, the spin polarization was set to +2 for one fragment and −2 for the other to ensure antiparallel spins during EDA. Orbital plots and graphics were visualized and generated with ChemCraft^78^,

## Supporting Information Available

Visualizations of investigated structures, molecular orbitals, spin densities, additional EDA, and computational details (PDF)
Cartesian coordinates of DFT-optimized structures (XYZ)

## Supplementary Material

om3c00398_si_001.pdf

om3c00398_si_002.xyz
