# Supplementary material for: Is Mn(I) More Promising Than Fe(II)—A Comparison of Mn vs Fe Complexes for Olefin Metathesis
Source: Organometallics. 2024 Feb 5;43(4):457–66. doi: 10.1021/acs.organomet.3c00398 (PMC10900517; doi:10.1021/acs.organomet.3c00398)
Supplement: Supplementary file 1 — om3c00398_si_001.pdf [file om3c00398_si_001.pdf]

Supporting Information

Is Mn(I) more promising than Fe(II)-- A comparison of Mn vs. Fe complexes for olefin metathesis

Jan Pecak,[†] Radu A. Talmazan,[†] Dennis Svatunek,[‡] Karl Kirchner,[‡] and Maren Podewitz[†]*

[†] Institute of Materials Chemistry, TU Wien, Getreidemarkt 9, 1060 Vienna, AUSTRIA. [‡] Institute of Applied Synthetic Chemistry, TU Wien, Getreidemarkt 9, 1060 Vienna, AUSTRIA.

*Email: maren.podewitz@tuwien.ac.at

[Mn(NHC)(CO)$_2$(C$_2$H$_4$)(CH$_2$)] (1-Mn)

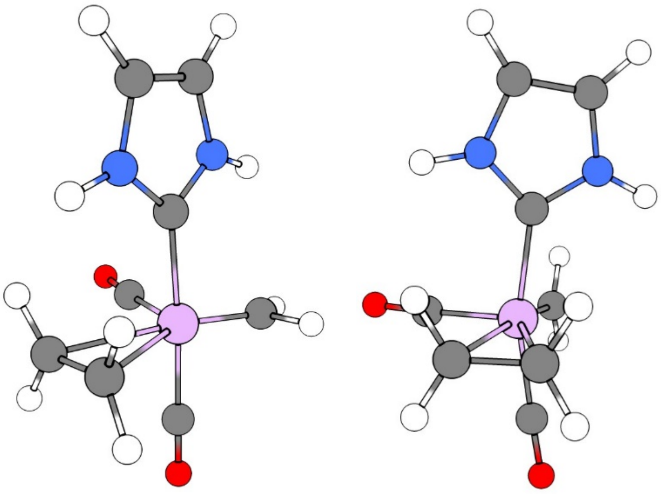

Fig. S1. Adduct [Mn(NHC)(CO)$_2$(C$_2$H$_4$)(CH$_2$)]$^+$ in S = 0 (left) and S = 1 (right) state.

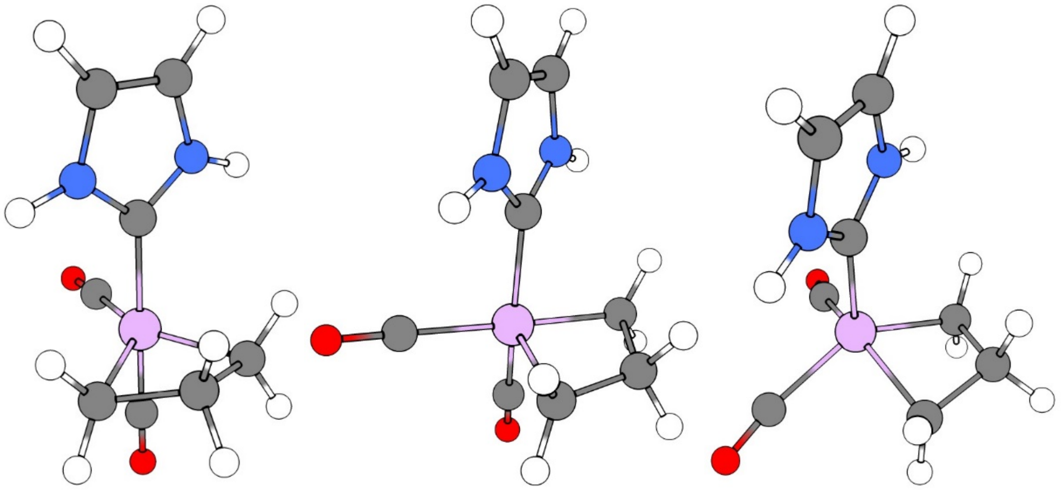

Fig. S2. Metallacycles [Mn(NHC)(CO)$_2$(C$_3$H$_6$)]$^+$ in S = 0, S = 1 and S = 2 state (left, center, right).

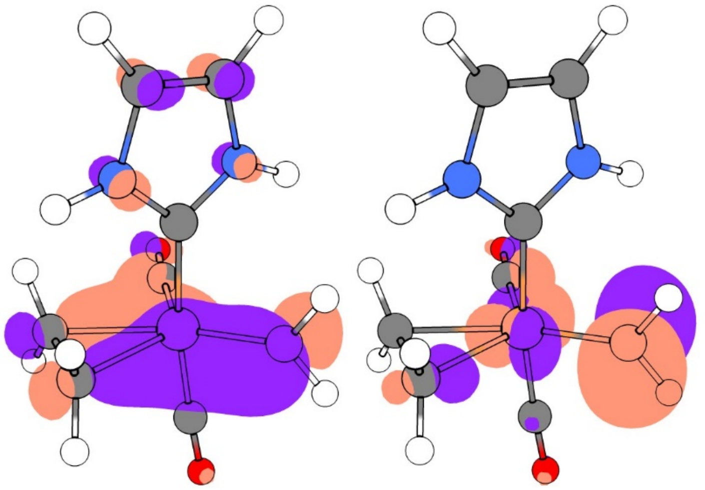

Fig. S3. HOMO and LUMO of [Fe(NHC)(CO)$_2$(C$_2$H$_4$)(=CH$_2$)] (**1-Fe**) (isovalue = 0.05).

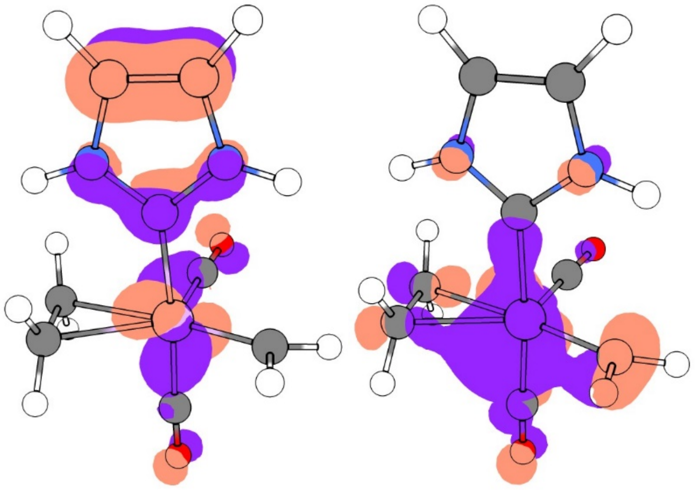

Fig. S4. HOMO and LUMO of $[Mn(NHC)(CO)_2(C_2H_4)(=CH_2)]^+$ (**1-Mn**) (isovalue = 0.05).

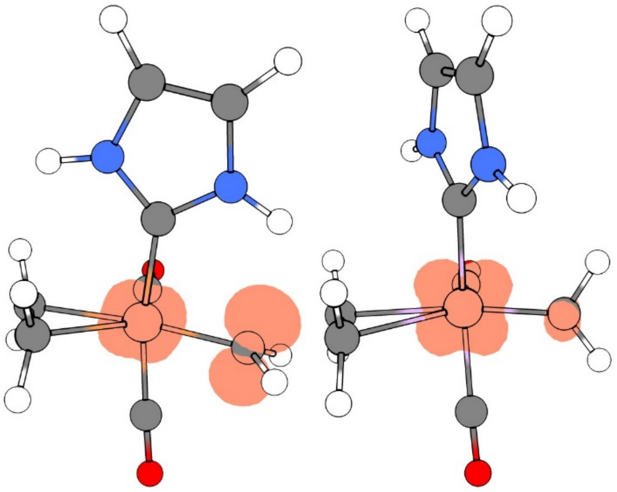

Fig. S5. Spin densities (S = 1) of the **1-Mn** and **1-Fe** olefin adducts (isovalue = 0.015).

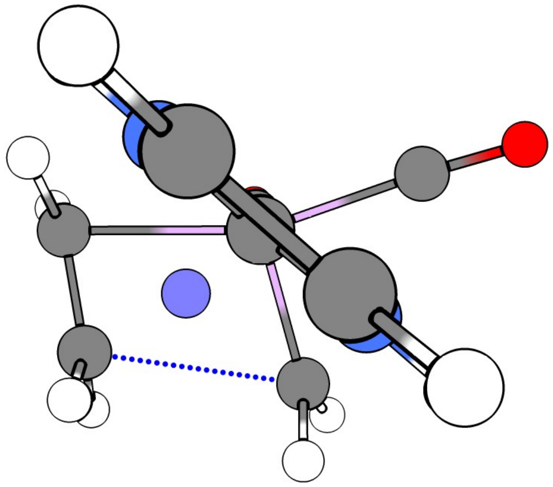

Fig. S6. Definition of reference coordinate point for NICS(1) in singlet $TS_{[2+2]}$ structure. The blue ghost atom is located 1.00 Å above the plane.

[Mn(PCP)(CH_2)] and [Mn(PCP)(C_3H_6)] (2-Mn)

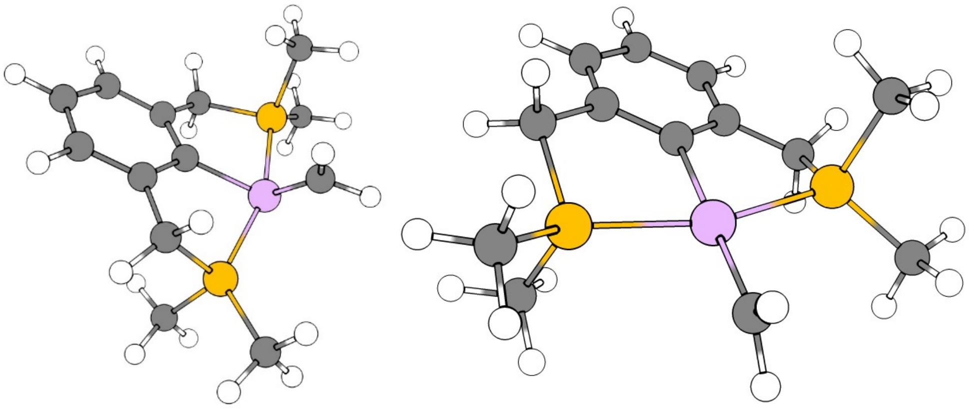

Fig. S7. Initial metal carbene complexes in the S = 0 and S = 1 state.

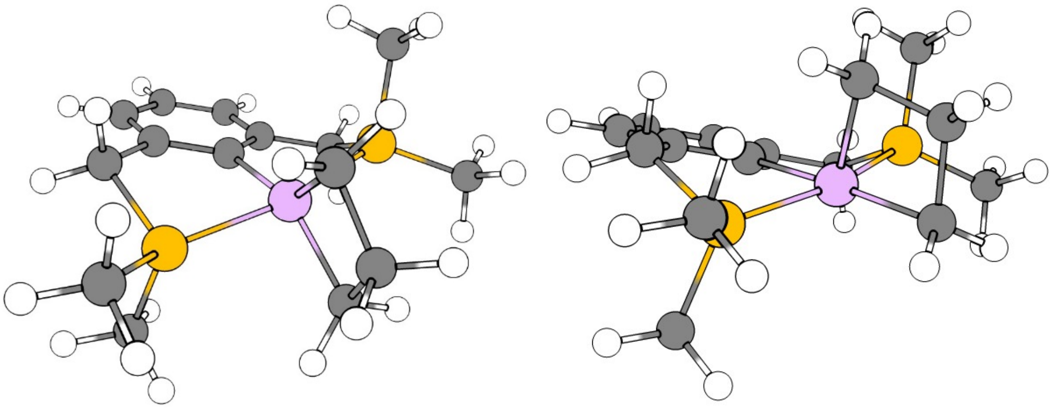

Fig. S8. Metallacyclobutane intermediates in S = 0 and S = 1 state.

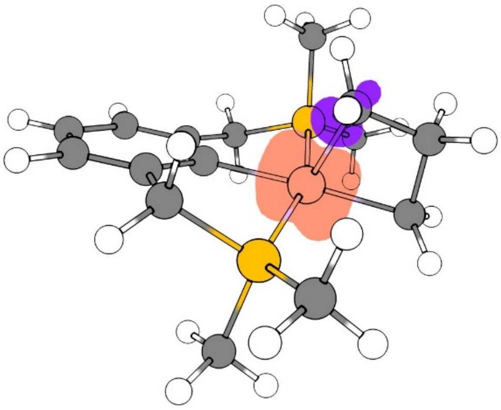

Fig. S9. Spin density (S = 1) of metallacycle [Mn(PCP)(C_3H_6)] (isovalue = 0.015). The mulliken spin population on Mn = 2.34 and on C(α) = –0.24.

Ruthenium systems for comparison

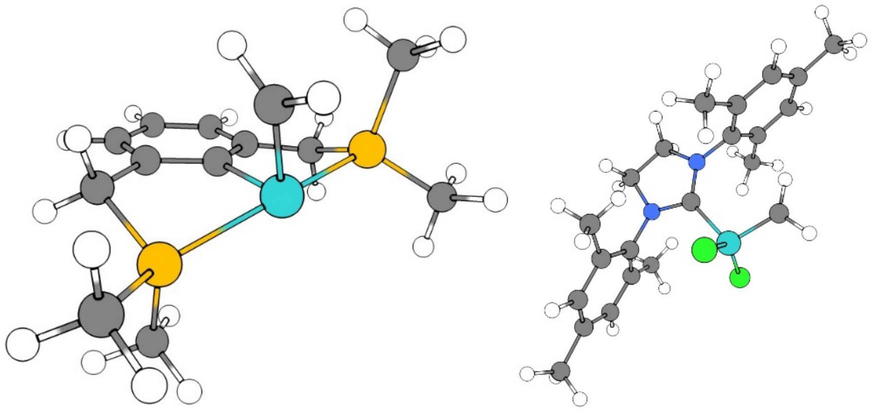

Fig. S10. Ruthenium analogue (**2-Ru**) to carbenes **2-Mn**/**2-Fe** and **[Ru(IMesH$_2$)(=CH$_2$)Cl$_2$]**.

Energy decomposition analysis for 2-Ru and [Ru(IMesH$_2$)(=CH$_2$)Cl$_2$].

For comparison with Ruthenium based systems, a structurally analogous system to **2-Mn/2-Fe** was used (**2-Ru**), as well as a the well-known Grubbs System **[Ru(IMesH$_2$)(=CH$_2$)Cl$_2$]**. The CH$_2$ unit was used as second fragment in the EDA analysis using ADF. See Computational Methods for further details. For both compounds a “electron-sharing” bonding mode is more favorable, similar to **2-Mn**.

Table S1. EDA for complexes **2-Ru** and **[Ru(IMesH$_2$)(=CH$_2$)Cl$_2$]**. Energy values are given in kcal/mol obtained at TPSSh/TZ2P/D3.

	Bonding mode	ΔE_{int}	ΔE_{elstat}	ΔE_{orb}	ΔE_{Pauli}
**2-Ru**	Dative	-147.0	-306.2	-215.7	379.9
**[Ru(IMesH$_2$)(=CH$_2$)Cl$_2$]**		-130.1	-380.8	-322.4	578.8
**2-Ru**	Electron-sharing	-106.7	147.0	-182.3	227.8
**[Ru(IMesH$_2$)(=CH$_2$)Cl$_2$]**		-98.7	-158.4	-172.6	237.9

Influence of exchange-correlation functionals on the singlet-triplet splitting in complex 1.

The metallacycle is the most crucial intermediate in first row transition metal olefin metathesis. To study the influence of the exchange correlation functional on the relative spin state splitting, single point calculations were performed using the def2-TZVP basis set on the previously obtained geometries. It is relevant to notice that implicit solvation (PCM, dichloromethane) used has a significant effect on the singlet-triplet splitting as was already reported by Shirazi et al. [1] and Gonzalez et al. [2] for other systems. In the present case a reduction of ΔE_{ST} by approx. 2 kcal/mol can be observed. On the other hand, solvation is necessary to stabilize the cationic systems in our study.

For reference purposes, single point calculations were also carried out with DLPNO-CCSD(T) utilizing an unrestricted BP86 reference wave function. The "T1" diagnostic values were in both cases < 0.018. Clearly, TPSSh yielded the best agreement with the DLPNO-CCSD(T) reference data.

Table S2. Relative spin-state splitting for the metallacycle of complex **1-Mn**. The energy values are given in kcal/mol.

Functional	ΔG_{ST} [kcal/mol]
TPSSh	–5.7
B3LYP	–12.4
OPBE	–1.7
TPSS	–1.0
BP86	–1.1
B2PLYP	12.6
PWPB95	6.3
DLPNO-CCSD(T)	–5.4

Table S3. Relative spin-state splitting ΔE_{ST} for the metallacycle of complex **2-Mn** and **2-Fe.** The energy values are given in kcal/mol.

Functional	2-Mn	2-Fe
TPSSh	–5.5	0.8
B3LYP	–12.1	–6.1
OPBE	–10.5	5.2
TPSS	0.6	8.0

Minimum Energy Crossing Points

MECP were located using the algorithm implemented in ORCA using the SurfCrossOpt and NoTRAH keywords. The singlet metallacycle geometry was used as a starting point. The nature of the MECP was additionally verified by a numerical Hessian calculation invoked using the SurfCrossNumFreq keyword. To obtain free energies corrected for low frequencies, an approximation was used adding one half of the calculated thermal and entropic corrections of the singlet and triplet metallacycle, each.

NICS and NMR shielding

For the calculation of NMR shifts the GIAO (Gauge Including Atomic Orbitals) method was used as implemented in ORCA by default. Isotropic 13-Carbon shifts (in ppm) are given relative to TMS. Values for the reference molecule were obtained at the same level of theory (TPSSh/def2-TZVP). To calculate the NICS values to investigate the aromaticity of the TS, a ghost atom (" H: ") was placed in the geometric center of the TS ring structure (see Fig. S6).

References

[1] R. G. Shirazi, F. Neese, D. A. Pantazis *Journal of Chemical Theory and Computation* **2018**, 14, 4733-4746.

[2] C. Gonzalez, A. Restrepo-Cossio, M. Márquez, K. B. Wiberg, M. De Rosa *The Journal of Physical Chemistry A* **1998**, 102, 2732-2738.
